# Extracellular Vesicle-Mediated Chemoresistance in Oral Squamous Cell Carcinoma

**DOI:** 10.3389/fmolb.2021.629888

**Published:** 2021-03-09

**Authors:** Zhu-Jun Law, Xin Hui Khoo, Pei Tee Lim, Bey Hing Goh, Long Chiau Ming, Wai-Leng Lee, Hui Poh Goh

**Affiliations:** ^1^ School of Science, Monash University Malaysia, Selangor, Malaysia; ^2^ College of Pharmaceutical Sciences, Zhejiang University, Hangzhou, China; ^3^ Biofunctional Molecule Exploratory (BMEX) Research Group, School of Pharmacy, Monash University Malaysia, Selangor, Malaysia; ^4^ PAP Rashidah Sa’adatul Bolkiah Institute of Health Sciences, Universiti Brunei Darussalam, Gadong, Brunei Darussalam

**Keywords:** oral squamous cell carcinoma, extracellular vesicles, chemotherapy, chemoresistance, oral cancer

## Abstract

Oral Squamous Cell Carcinoma (OSCC) remains a cancer with poor prognosis and high recurrence rate. Even with multimodal treatment options available for OSCC, tumor drug resistance is still a persistent problem, leading to increased tumor invasiveness among OSCC patients. An emerging trend of thought proposes that extracellular vesicles (EVs) play a role in facilitating tumor progression and chemoresistance via signaling between tumor cells. In particular, exosomes and microvesicles are heavily implicated in this process by various studies. Where primary studies into a particular EV-mediated chemoresistance mechanism in OSCC are limited, similar studies on other cancer cell types will be used in the discussion below to provide ideas for a new line of investigation into OSCC chemoresistance. By understanding how EVs are or may be involved in OSCC chemoresistance, novel targeted therapies such as EV inhibition may be an effective alternative to current treatment options in the near future. In this review, the current understandings on OSCC drug mechanisms under the novel context of exosomes and microvesicles were reviewed, including shuttling of miRNA content, drug efflux, alteration of vesicular pH, anti-apoptotic signaling, modulation of DNA damage repair, immunomodulation, epithelial-to-mesenchymal transition and maintenance of tumor by cancer stem cells.

## Introduction

Oral cancer, categorized under head and neck cancer, consists of cancers occurring in the oral cavity. Cancers originated from oral cavity, tongue, lip and mouth accumulatively represents the 8th most common cancer with more than 300,000 cases per annum (D'Cruz et al., 2018; Elaiwy et al., 2020). There were roughly about 65,410 head and neck cancers cases recorded in the United States in 2019, which is accountable for 3.7% of new cancers (Kim and Kim, 2020). Besides, the incidence and mortality rate for OSCC is regional and its prognostic is still unfavourable (Sim et al., 2019). OSCC is highly mortal despite occurring in a fairly accessible location: the 5-year survival rate stands at only 47–66% as most oral cancer cases were detected at a late stage of malignancy (Siegel et al., 2017). More than 90% of cases with oral cancer are classified as squamous cell carcinoma (Leite et al., 2018). Oral squamous cell carcinoma (OSCC) originated from oral keratinocytes. The main risk factors known to cause oral cancer include tobacco smoking and alcohol consumption, while other mutagens such as betel quid chewing, diets of low fruit and vegetable content, and infections are known to play a causative role in some oral cancer cases as well (Zain, 2001; Llewellyn et al., 2004; Bunnell et al., 2010; Alnuaimi et al., 2015). Exposure to mutagenic substances can cause spontaneous mutations which induce persistent inflammation in the cells, leading to the development of precancerous lesions. If left untreated, these precancerous lesions (known as leukoplakia and erythroplakia) may further develop into cancerous cells (Belcher et al., 2014).

Current treatments for oral cancer include surgery, chemotherapy, radiotherapy and immunotherapy (Gharat et al., 2016). Depending on the stages of the cancer malignancy, different treatment approaches will be applied. For the early stages, cancer patients are typically treated with either surgery or radiotherapy, and patients with an advanced stage of cancer receive treatment with a combination of surgery and radiation with or without chemotherapy (Belcher et al., 2014). Incorporated multimodality treatment is employed to fully eradicate possible unremoved cancer cells from the first treatment method and has shown to improve the overall survival of OSCC patients (Furness et al., 2011). However, most patients will inevitably experience tumor progression or disease recurrence which usually accompanied with an increase of tumor invasiveness. As reported in many clinical studies, the rate of OSCC recurrence ranged from 18 to 76%, and the majority occurred within 2 years post treatment completion (Myers et al., 2001; Carvalho et al., 2005; Wang et al., 2013). The treatment choices for recurrent OSCC cases are limited; more than 80% of loco-regional recurrences which happen within the first two years are highly associated with poor prognosis when undergoing salvage surgery (Kernohan et al., 2010). The factors influencing recurrence rate could be due to how advanced the disease is when discovered, development of cells with acquired drug resistance, or the emergence of cell subpopulations genetically refractory to the drugs (Da Silva et al., 2012).

Current research on OSCC drug resistance centers around elimination and inactivation of drug from cancer cells, increased response to DNA damage, reduced apoptosis and increased migratory abilities of cancer cells (Wang et al., 2016).These drug resistance mechanisms are not unique to just OSCC but are shared across different types of cancer cells. An emerging trend of thought is the involvement of extracellular vesicles in mediating these drug resistance mechanism (Khoo et al., 2019). Extracellular vesicles specifically exosomes and microvesicles have been heavily implicated in cancer drug resistance by various studies (Yu et al., 2016; Khoo et al., 2019; Steinbichler et al., 2019). The aim of this review is to re-examine OSCC drug resistance mechanisms with the novel lens of extracellular vesicles.

### Chemoresistance in OSCC Cells

Chemoresistance—the resistance of cancer cells to drugs used in chemotherapy—is a major impediment in cancer treatment as it causes long term poor prognosis and increases the chance of metastasis. There are mainly two types of drug resistance in cancer tumors, which are *de novo* drug resistance and acquired drug resistance. *De novo* drug resistance is present before drug exposure and selection for drug resistance, while acquired drug resistance, also known as adaptive drug resistance refers to resistance that is developed over time after prolonged exposure to chemotherapy drugs (Hazlehurst and Dalton, 2006). *De novo* drug resistance arises before drug exposure due to accumulating mutations over time. Some of these mutations may have a selective advantage during chemotherapeutic treatment (Friedman, 2016). Acquired drug resistance has been modeled in tissue culture by chronic exposure to a cytotoxic agent, until a stable drug resistance phenotype is selected. Upon treatment, a pre-existing mutation that carries a selection benefit to the treated tumor cells becomes fixed in the population. The longer the treatment, the higher the likelihood a resistance mutation will be fixed (Friedman, 2016). Moreover, other adaptive responses, such as decreased expression of the therapeutic target and activation of alternative compensatory signaling pathways may arise during treatment, contributing to adaptive resistance (Longley and Johnston, 2005). As such, the intuition that an effective chemotherapeutic drug eliminates the bulk of cancer cells and induces short-term remission may be misleading as the elimination process may effectively select for a chemoresistant subpopulation.

**FIGURE 1:**
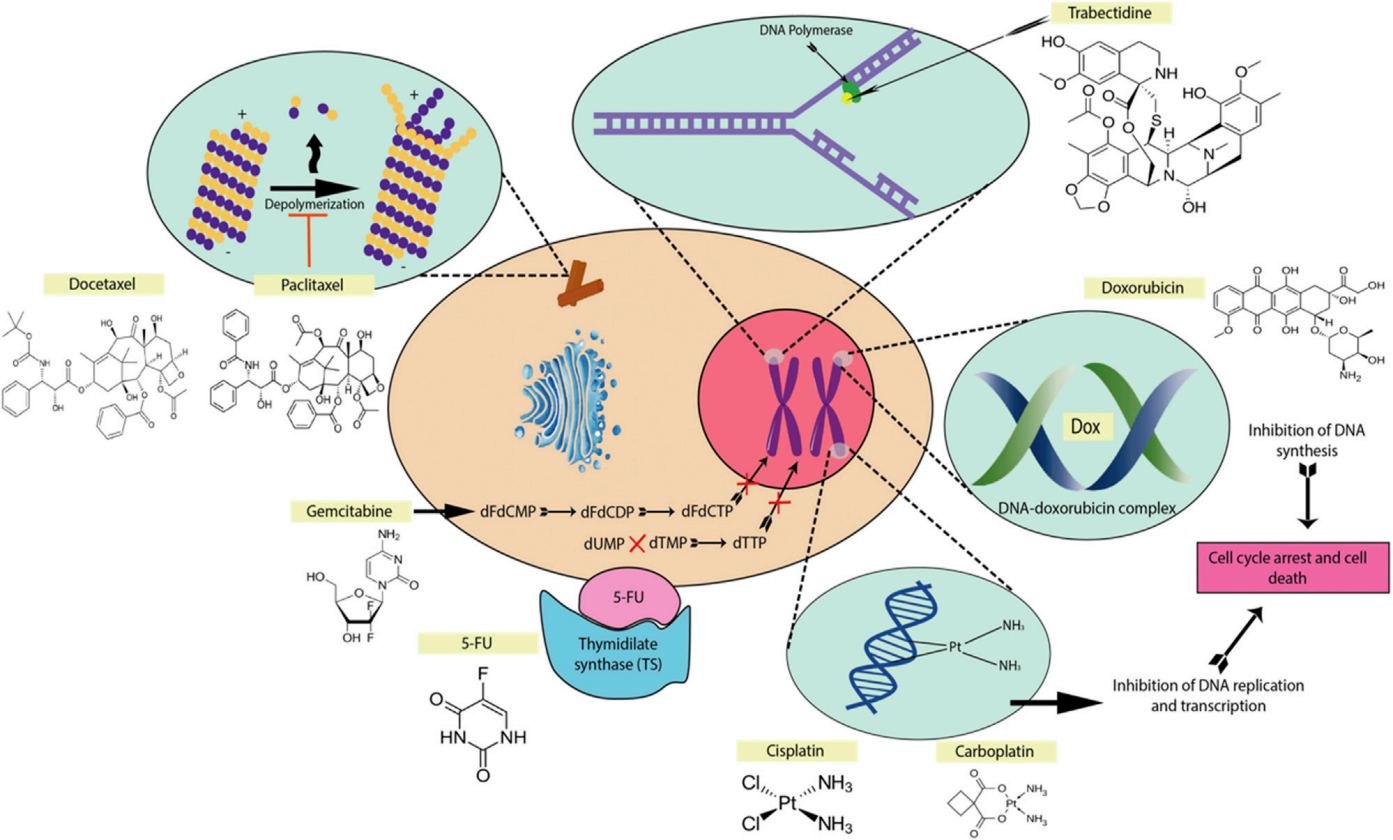
The mechanisms of action of some common chemotherapeutic drugs. The main mechanisms are forming DNA crosslinks (cisplatin, carboplatin), disrupting topoisomerase-II-mediated DNA repair (doxorubicin), promote microtubule polymerization and stabilization (paclitaxel, docetaxel), binding to the minor groove of DNA (trabectedin), inhibiting thymidylate synthase (5-fluorouracil), antimetabolite for pyrimidine nucleoside (gemcitabine) (Larionova et al., 2019).

Chemotherapy drugs which are used for the treatment of OSCC include platinum-based drug e.g., like cisplatin and carboplatin, taxanes like paclitaxel and docetaxel, anthracyclines such as adriamycin, epirubicin, pirarubicin, doxorubicin and antimetabolites such as methotrexate and 5-fluorouracil (5-FU) (Figure 1). They often work by inducing molecular cascades which result in cell cycle arrest or cell death in cancerous tumors. When a chemotherapy drug directly or indirectly induces damage to DNA, a mechanism known as the DNA damage response (DDR) is activated to coordinate various pathways which result either in DNA repair and cell cycle arrest or apoptosis of damaged cells (Helena Lobo et al., 2007). Chemotherapy drugs such as paclitaxel and docetaxel act by stabilizing microtubules, causing a G2M arrest and later inducing apoptosis (Shah and Schwartz, 2001). On the other hand, platinum-based drugs acts as a DNA intercalating agent and is able to cause DNA damage directly, which leads to the activation of cyclin-dependent kinase inhibitors (CDKIs) and inducing cell cycle arrest in the G2 phase (Sorenson and Eastman, 1988). Anthracyclines such as doxorubicin intercalate between DNA base pairs and inhibits topoisomerase II essential in resolving supercoiling during DNA replication. In addition, antimetabolites such as methotrexate and 5-FU inhibit the action of thymidylate synthase, preventing dTTP production and DNA replication.

Platinum-based chemotherapy drugs which are commonly used in combination with 5-FU are still the usual first-line treatment for OSCC, but the results are far from satisfactory. In advanced OSCC cases, chemotherapy drugs such as methotrexate, paclitaxel and docetaxel are more commonly used either alone or in combination (Specenier and Vermorken, 2010). Despite initial significant results in the survivability OSCC patients, these treatments ultimately fail due to the development of chemoresistance.

## Role of Extracellular Vesicles in OSCC Chemoresistance

Extracellular vesicles (EVs) are lipid-bilayer enclosed vesicles that are naturally released from cells. Over the last decade, intercellular communication mediated by EVs present a new paradigm in which chemoresistance mechanisms should be investigated. Unlike traditional cell-cell signaling via cytokines, chemokines and growth factors (Ahmed and Xiang, 2011), EVs contain cellular cargoes such as miRNAs, lncRNA and proteins that may be endocytosed by other recipient cells. Accumulating evidence points to these cargoes mediating drug resistance properties from cell-to-cell in various cancer types.

EVs can be differentiated into three main categories: exosomes (30–100 nm), microvesicles (MVs) (100–1,000 nm), and the more recently discovered ‘oncosomes’ (1–10 μm) (Minciacchi et al., 2015). These categories are also differentiated by their mechanism of biogenesis and cellular origins. It is also of importance to note that EV size distinctions are not absolute (i.e. MVs may be <100 nm) and methods to distinguish between exosomes and MVs remain ambiguous from laboratory to laboratory. Since most studies of EV-mediated chemoresistance in OSCC have been conducted on exosomes and MVs, the main focus below will be on these two categories.

### Exosomes

Exosomes are the smallest subset of extracellular vesicles secreted by cells. Their sizes (30–100 nm) vary depending on the cellular source or sample isolation and preparation methods (Wu et al., 2015). They were first discovered in maturing mammalian reticulocytes during the golden era of electron microscopy. It was observed that maturing reticulocytes contained large sacs filled with small membrane enclosed vesicles of nearly uniform size (30–100 nm) within their cytoplasm (Johnstone, 2005). In both normal cells and cancer cells, exosomes play a role in the removal of unwanted materials from the cell and communication between cells via the transfer of bioactive molecules packaged into the exosomes. The efflux of exosomes is one way of eliminating cell waste products–the other way being lysosomal degradation (autophagy) (Baixauli et al., 2014).

**FIGURE 2:**
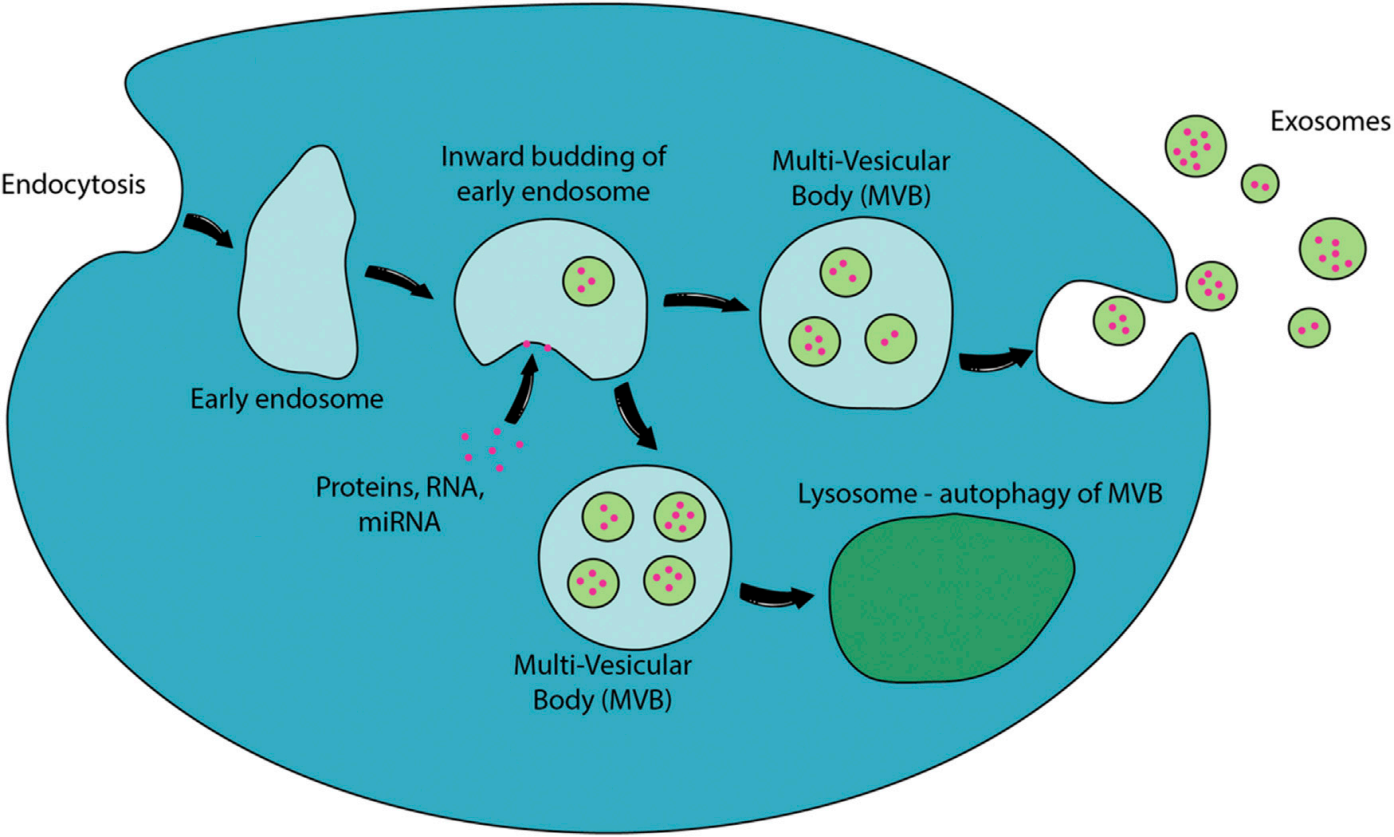
Biogenesis of exosomes. EVs are released to the extracellular environment through direct outward budding or “pinching” of the cellular membranes. Proteins, RNA, miRNA or other potential exosomal contents will be packed into early endosomes. The inward budding of the early endosomes to form intraluminal vesicles and the fusion of multi-vesicular body (MVB) with plasma membrane releases exosomes to the extracellular environment. MVB could also fuse with lysosomes which will degrade their internal contents through autophagy (Raposo and Stoorvogel, 2013).

Exosomes originate from the early endosome which accumulates intraluminal vesicles (ILVs) formed through the inward budding of its membrane (Colombo et al., 2014). The endosomes are referred to as multivesicular bodies (MVBs) due to the multiple ILVs contained in the endosomes. During the inward budding of the early endosome, proteins, lipids and RNAs are selectively sorted into ILVs. The MVBs containing loaded ILVs are either fused with lysosome for degradation or fuse with the plasma membrane for secretion–the secreted ILVs are referred to as exosomes (Colombo et al., 2014). The overall biogenesis of exosomes is presented in Figure 2.

This entire process can either be driven by the endosomal sorting complexes required for transport (ESCRT) or by other ESCRT-independent mechanisms such as neutral sphingomyelinase-dependent ceramide formation. Exosomes secreted from one cell can fuse with the plasma membrane of another cell and can be internalized through endocytosis or pinocytosis, therefore, transferring bioactive molecules from one cell to another (Théry et al., 2002). The exosomal contents that are transferred to other cells can affect the function of the recipient cell. For example, the transfer of exosomal RNA from antigen-presenting dendritic cells can stimulate proliferation in T lymphocytes (Segura et al., 2005). In cancer cells, exosomes play important roles in tumorigenesis, growth, progression, metastasis and drug resistance (Liu et al., 2017).

#### Exosome-Mediated Chemoresistance

Exosomes play many important roles in enabling drug resistance in cancer cells through multiple mechanisms such as shuttling of miRNA, drug efflux, anti-apoptotic signaling etc. In order to understand the effect of exosomes in both donor and recipient cells, it is imperative to investigate the exosomal cargo being shuttled between tumor cells.

##### Exosomal Content (MiRNAs)

Due to the exosome’s ability to transfer diverse loads of proteins, lipids and nucleic acids between cells, they have been dubbed as ‘Trojan horses’ (Gould et al., 2003). Exosomes secreted by cancer cells contain various functional nucleic acids such as circular DNA, mRNA, microRNAs (miRNAs), long noncoding RNAs (lncRNAs), transfer RNAs (tRNAs), small nucleolar RNAs (snoRNAs), ribosomal RNAs (rRNAs) and small nuclear RNAs (snRNAs) (Gajos-Michniewicz et al., 2014). DNA fragments and mutated mRNA transcripts are involved in the progression and angiogenesis of primary and metastatic cancers (Skog et al., 2009; Dvořáková et al., 2013). Cancer-derived exosomes show specific miRNA and mRNA expression profiles which vary according to the type of cancer (Ming-Hui et al., 2015; Yousafzai et al., 2018).

There is a significant body of evidence supporting the theory that exosomes, in their capacity to act as agents of intercellular communication, disperse the characteristic of drug resistance between tumor cells by delivering miRNAs (Zhang and Wang, 2017). MicroRNAs are small single-stranded highly conserved non-coding RNAs of 20–25 nucleotide length that are involved in post-transcriptional regulation of gene expression. The main function of miRNA is to base-pair with target mRNA to negatively regulate gene expression (Bartel, 2004; Macfarlane and Murphy, 2010). Regulated by cofactors, miRNAs also play a role in the activation of mRNA translation to regulate protein levels (Saraiya et al., 2013). Aside from that, they play a major role in controlling various activities in cells, including cell differentiation, proliferation, stress response, metabolism, cell cycle, apoptosis, and angiogenesis thus playing roles in the regulation of many diseases, including cancers (Jiang et al., 2009). Table 1 outlines various miRNAs that are implicated in OSCC chemoresistance along with its cellular effects.

**TABLE 1:** Roles of miRNA in regulation of drug resistance of OSCC.

MicroRNAs	OSCC Cell lines	Effect	References
*miR-21*	Tca8113/DDP	Regulation of STAT3 expression	(Xuan et al., 2014)
	HSC-3-R	Confers cisplatin resistance by targeting PTEN and PDCD4	(Liu et al., 2017)*
	CAL27	Reprogram monocytes via NF-*κ*B pathway	(Momen-Heravi and Bala, 2018)*
*miR-24*	CAL27, HSC6	Reduce PTEN expression	(Zheng et al., 2015)
	SCC25, HSC6	Binds to PER1 and induces cell proliferation and cell cycle progression	(He et al., 2020)*
*miR-29a-3p*	SCC9 and CAL27	Promote M2 subtype macrophage polarisation	(Cai et al., 2019)*
*miR-130b, miR-134, mi491, mi-149*	SCC131/R, SCC084/R	Modulation of EMT and metastasis	(Ruma Dey et al., 2016)
*miR-155*	SCC131	Downregulates FOXO3 and promotes EMT phenotype	(Kirave et al., 2020)*
*miR-221*	Tca8113, UM2	Reduce TIMP3 expression	(Chen et al., 2016)
*miR-222*	UM1	Reduce PUMA expression	(Jiang et al., 2014)
*miR-1246*	SAS, GNM	Enhancing cancer stemness and chemoresistance via targeting CCNG2	(Shih-Shen et al., 2018)

STAT3: Signal transducer and activator of transcription 3; PTEN: Phosphatase and tensin homolog; PDCD4: Programmed cell death protein 4; NF-κB: Nuclear factor-κB; EMT: Epithelial-Mesenchymal Transition; TIMP3: Tissue inhibitor of metalloproteinase 3; PUMA: p53 activates transcription of p53-upregulated modulator of apoptosis; CCNG2: Cyclin-G2.

‘*’ Represents published literature which demonstrated that the respective miRNA was present in OSCC-derived exosomes.

A particular miRNA which has been reported widely in modulating cancer drug resistance is miR-21. Although the precise mechanism of action of miR-21 in OSCC has yet to be fully described, miR-21 is regarded as a key oncogenic factor that is associated with poor cancer prognosis and are highly expressed in tissues and blood of OSCC patients when compared to healthy tissues and blood control (Chang et al., 2008; Wong et al., 2008; Ren et al., 2014). MiR-21 was shown to be involved in the regulation of drug resistance in various types of cancers including renal cancer, breast cancer, leukemia cancers, glioblastoma, gastric cancer and many other cancer cell lines (Hong et al., 2013; Yang et al., 2013; Gaudelot et al., 2017). It has been confirmed that miR-21 induces chemoresistance by targeting PDCD4 and PTEN, two tumor suppressor genes (Wei et al., 2016; Liu et al., 2017). PDCD4 is involved in apoptosis and inhibits tumor progression by acting on eIF4A and eIF4G, suppressing mRNA translation (Liu et al., 2017) whereas PTEN diminishes the P13K/Akt/mTOR signaling pathway which results in tumor growth. It was also shown in the study by Liu et al. that (previously chemosensitive) OSCC cells developed resistance after being exposed to exosomes derived from resistant OSCC cells (which contained miR-21) (Liu et al., 2017).

The mechanism of miR-21 in OSCC drug resistance may also be through the regulation of STAT3, a transcription factor activated by IL-6. MiR-21 is known to affect STAT3 to promote tumor cell survival, induce anti-apoptotic effect, and also aid in cell cycle progression and angiogenesis (Iliopoulos et al., 2010; Xuan et al., 2014). The suppression of STAT3 with a specific inhibitor was shown to downregulate miR-21 in OSCC cells and sensitized cisplatin-resistant OSCC cells to cisplatin treatment (Xuan et al., 2014). This result supports the role of miR-21 in moderating cisplatin resistance in OSCC and suggests that STAT/miR-21 pathway can serve as a potential therapeutic target to develop chemosensitivity in cisplatin-resistant OSCC cells.

MiR-24 is another highly upregulated miRNA in OSCC tissues. It regulates a myriad of cellular activities and was reported to induce cell survival and cisplatin resistance in tongue squamous cell carcinoma when up-regulated (Zheng et al., 2015). Frequent dysregulation of miR-24 was observed in OSCC cells; upon further analysis, it was proven that miR-24 directly inhibits PTEN (a tumor suppressor gene) expression, resulting in the activation of the PI3K/Akt cell survival pathway and promoting cell cycle progression (Zheng et al., 2015). Loss-of-function mutations of PTEN are known to drive the resistance of cancer cells to many anticancer drugs (Dillon and Miller, 2014). Knockdown of miR-24 was shown to restore PTEN expression and resensitize cisplatin-resistant OSCC cells to cisplatin treatment (Zheng et al., 2015). Other targets of miR-24 besides PTEN include DND1 (regulates CDKN1B suppression which enhanced proliferation and reduced apoptosis in toungue SCC cells) (Liu et al., 2010), and more recently discovered PER1 (enhances cell proliferation and cell cycle progression of OSCC cells) (He et al., 2020). It is interesting to note that He et al., also proven that miR-24 is a cargo of salivary exosomes derived from OSCC patients. The increased cell proliferation and reduced apoptosis mediated by miR-24 counteracts against chemotherapeutic drug mechanisms such as cell cycle inhibition and DNA damage-induced apoptosis. Interestingly, miR-24 also have anti-tumour characteristics in other cancer types via FOXM1 which suppresses bladder cancer cell proliferation (Inoguchi et al., 2014) and LPAAT which suppresses osteosarcoma cell proliferation (Song et al., 2013). The diverse binding targets of miR-24 in different cancer types prompts further investigation to elucidate the complete pathways that miR-24 is involved in OSCC. This is crucial to increase the feasibility of proposed strategies that therapeutically targets miRNA in OSCC tissues. Without sufficient information of miRNA binding targets, it becomes difficult to predict cellular effects from upregulating or downregulating miRNA in OSCC tissues.

The discovery of miRNA involvement in chemoresistance is still novel and studies relating to OSCC are minimal at the moment. Moreover, one miRNA may target various tumor-related gene transcripts and the activity of one gene transcript may be regulated by multiple miRNAs. Thus, it is challenging to design therapeutic strategies which can efficiently eliminate drug resistance via a single miRNA (Hong et al., 2013). There are recent studies detailing the discovery of ‘master miRNAs' which target multiple drug resistance pathways (Manabu et al., 2017). This may be an alternative solution in treating OSCC drug resistance and requires further investigation. Aside from that, studies that prove the above discussed miRNAs to be present in OSCC-derived exosomes are limited. It is important to note that two populations of extracellular miRNAs exist in biological fluids–one found in vesicles such as exosomes and microvesicles; the other is associated with proteins such as Argonaute (AGO) (Turchinovich et al., 2011). There is a significant number of studies that do not investigate the origins of their respective OSCC-associated miRNAs. Therefore, the role of exosomes in conferring chemoresistance traits via these miRNAs have to be viewed with skepticism.

##### Drug Efflux

Multidrug resistance (MDR) is a phenotype in which cancer cells exhibit simultaneous resistance to multiple chemotherapeutic drugs. Overexpression of drug efflux transporters of the ATP binding cassette (ABC) transporter family such as P-glycoprotein (P-gp) and copper efflux transporters gives rise to the MDR phenotype in tumor cells (Yoshizawa et al., 2007; Pérez-Sayáns et al., 2010). Although yet to be proven in OSCC cell lines, exosomes from resistant tumor cells can transfer P-gp to chemosensitive cells and confer chemoresistance. Lv et al. (2014) demonstrated that P-gp expression in sensitive MCF-7 breast cancer cells were higher after incubation with exosomes isolated from docetaxel-resistant MCF-7 cells (Lv et al., 2014). However, expression analysis of P-gp in OSCC cells via RT-PCR revealed that only 18 % of OSCC cases are P-gp-positive (Friedrich et al., 2004). P-gp expression in other tumor types such as salivary gland adenocarcinoma and colon carcinoma are markedly higher than in OSCC (Uematsu et al., 2001; Friedrich et al., 2004). Nevertheless, P-gp levels in recurrent OSCCs are higher compared to normal mucosa with lesions at different stages of tumorigenesis (Jain et al., 1997). Aside from P-gp, Multidrug Resistance Associated Protein 1 (MRP1) is also linked to the MDR phenotype as well. MRP1 expression levels measured via RT-PCR and immunohistochemistry revealed that 20–30 % of OSCC cases have higher than normal expression (Tsuzuki et al., 1998; Uematsu et al., 2001). These results show that the set of proteins giving rise to the MDR phenotype in OSCC cells is different compared to other tumor types.

Platinum-based antitumor drugs such as cisplatin and carboplatin are the mainstream chemotherapy drugs used clinically. Ctr1, the major copper influx transporter has been demonstrated to transport cisplatin and other analogues such as carboplatin and oxaliplatin. More evidence of two other copper efflux transporters—ATP7A and ATP7B are also accumulating (Komatsu et al., 2000; Kuo et al., 2007; Yoshizawa et al., 2007). Mechanisms for transporting platinum drugs were not known until evidence of it related to copper homeostasis began to build. Many cell lines that exhibited resistance to platinum drugs also showed cross-resistance to copper drugs and vice versa (Safaei et al., 2005). However, enhanced cisplatin intake via increased expression of copper influx transporter, Ctr1 does not sensitize tumor cells to cisplatin nor does it increase cisplatin-DNA adduct formation (Beretta et al., 2004; Holzer et al., 2004). One plausible reason is that Ctr1 does not deliver cisplatin in a way that allows it to access key intracellular cytotoxic targets. The two copper efflux transporters—ATP7A and ATP7B are located in the *trans*-Golgi network (TGN) and are involved in copper-stimulated trafficking (Didonato and Sarkar, 1997; Camakaris et al., 1999; Prohaska and Gybina, 2004). Under conditions of excess copper in the cytoplasm, ATP7A and ATP7B are transported from the TGN to the plasma membrane to facilitate copper efflux until copper levels return to normal homeostatic ranges (Hellman and Gitlin, 2002). It is not known if cisplatin levels affect the normal transportation of ATP7A and ATP7B from the TGN to the plasma membrane. Moreover, the vesicular transport pathways between TGN and endosomes are complicated and it is not known if ATP7A/B is transported to the endosomal membrane from the TGN in OSCC cells (Progida and Bakke, 2016).

**FIGURE 3:**
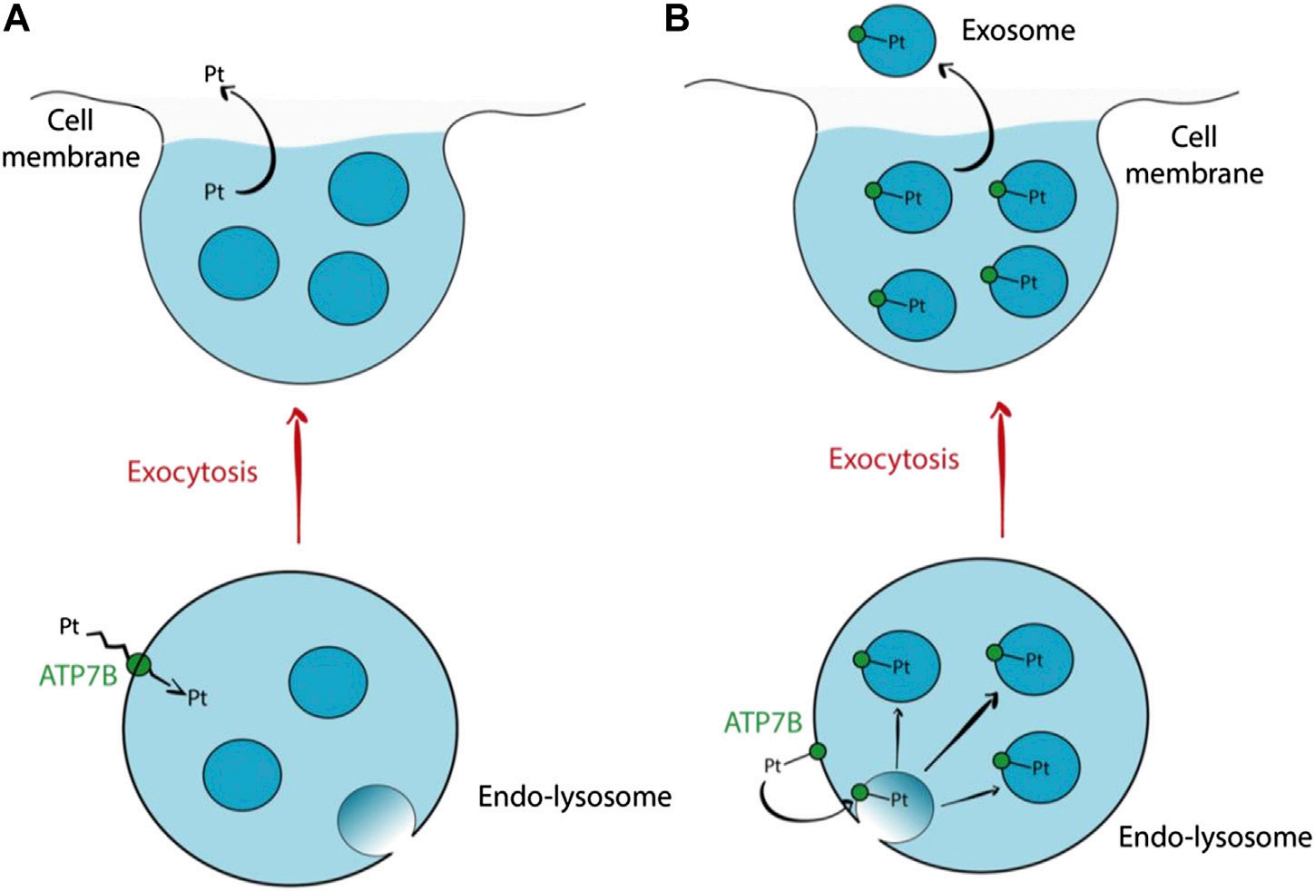
Hypothetical mechanisms of cisplatin efflux via lysosomal exocytosis involving ATP7B **(A)** ATP7B residing in the endo-lysosomal membrane transports cisplatin across the membrane into the endo-lysosomal lumen. Subsequent fusion of endo-lysosome with the cell membrane releases the cisplatin along with the ILVs. This proposed mechanism does not involve exosomes for cisplatin efflux. **(B)** Cisplatin is bound to ATP7B via the MBS. The ATP7B will then be incorporated into the membrane of a new ILV formed via budding of the endo-lysosomal membrane. The cisplatin will then follow the ILVs out from the cell when they are released as exosomes (Petruzzelli and Polishchuk, 2019).

Another school of thought supports the idea that cisplatin efflux via ATP7A/B involves lysosomal exocytosis (Petruzzelli and Polishchuk, 2019). ATP7A/B residing in the membrane of endo-lysosome (formed via fusion of endosome and lysosome) facilitates the influx of cisplatin into the lumen. Subsequent fusion of the endo-lysosome with the cell membrane releases cisplatin into the extracellular environment. Safaei et al*.* (2005) demonstrated that cisplatin resistant ovarian carcinoma cells have a lesser endo-lysosome volume and expression of lysosome-associated membrane proteins (LAMP1 and LAMP2), implying a marked increase in lysosomal exocytosis to expel cisplatin (Safaei et al., 2005). However, Safaei et al. (2005) also discovered that exosomes released from the resistant cells contain 2.6-fold more platinum than those released from sensitive cells (Safaei et al., 2005). An alternate mechanism of cisplatin efflux via lysosomal exocytosis involves intraluminal vesicles (ILVs). Transmembrane proteins such as ATP7A/B residing in the membrane of endo-lysosome can end up in new ILVs formed via budding of the endo-lysosomal membrane. Cisplatin are bound to the metal binding sites (MBSs) of ATP7A/B on the endo-lysosomal membrane which will be incorporated into the ILV membrane. The cisplatin will then be transported out from the cells when the ILVs are released as exosomes. Figure 3 below outlines both mechanisms of drug efflux involving ATP7B. Safaei et al. (2005) results support the latter mechanism as they reported a marked increase in exosomal levels of putative cisplatin transporters, mainly ATP7A and ATP7B (Safaei et al., 2005). Such a mechanism does not require the transport of cisplatin across a membrane, but rather requires cisplatin to be bound to the MBSs of ATP7A/B. Hence, it is likely a less energy-consuming mechanism of drug efflux.

Although results from Safaei et al. (2005) have yet to be proven true in OSCC cells, there is already evidence pointing toward ATP7B as a key contributor to cisplatin-resistance in OSCC cells (Safaei et al., 2005). Yoshizawa et al. (2007) demonstrated that resistant OSCC cell lines, namely HSC-4-R, OSC-19-R and HOC313-R all have higher expression of ATP7B (Yoshizawa et al., 2007). Transfection of ATP7B siRNA in OSC-19-R cells resensitized the cells to cisplatin by a factor of 10.6 (Yoshizawa et al., 2007). These results were not observed in ATP7A and Ctr1, pointing to a bigger role played by ATP7B in the context of OSCC cisplatin resistance. It is plausible that cisplatin efflux in OSCC cells may follow the lysosomal exocytotic pathway. However, experiments to measure the volume of lysosome in cisplatin-resistant and sensitive OSCC cells are needed to prove this theory. Furthermore, vesicular transport of ATP7B from TGN to endo-lysosome in OSCC cells remains unproven.

##### Alteration of Vesicular pH

The difference in pH between intracellular and extracellular pH plays an important role in the transport of chemotherapy drugs. Under normal circumstances, extracellular pH in OSCC is significantly more acidic than in normal tissue (Perez-Sayans et al., 2009). The acidic environment disrupts the absorption of chemotherapy drugs (Griffiths, 1991; Negendank, 1992). Studies conducted by Becelli et al. (2007) found that reversed pH gradient is an intrinsic tumor phenotype giving rise to drug resistance in oral cancer (Becelli et al., 2007). The intracellular and extracellular pH of oral cancer tumor specimens were found to be lower than normal tissues (Becelli et al., 2007).

It has been widely accepted that the changes in pH are mediated by vacuolar-ATPases (V-ATPases). V-ATPase is a multi-subunit ATP driven proton pump that regulates the intracellular and extracellular pH (Pamarthy et al., 2018). Aside from regulating the acidity of the tumor microenvironment from the plasma membrane (Perez-Sayans et al., 2009), V-ATPases are thought to play a role in vesicular acidification. Martínez-Zaguilán et al. was unable to trace V-ATPases in the plasma membrane via immunohistochemical analysis and concluded that V-ATPases are not statistically resident in the cell membrane of MCF-7 breast carcinoma cells (Martinez-Zaguilan et al., 1999). Resistant MCF-7 cells showed increased V-ATPase activity as they were capable of recovering from an acid load without anion exchangers (Martinez-Zaguilan et al., 1999). The failure to localize V-ATPase, coupled with the presence of V-ATPase activity suggests that the measured activity of V-ATPase might be the consequence of “rapid endomembrane turnover”. Thus, it can be hypothesized that V-ATPase resides more extensively on vesicle membrane rather than statically in the plasma membrane. This hypothesis is yet to be proven experimentally in OSCC cells. However, it suffices to note that V-ATPase is commonly localized in vesicular membranes of cancerous cells.

Raghunand et al. (1999) postulated that turnover of acidic vesicles is an important factor of chemoresistant cells, particularly those that do not overexpress P-gp efflux pumps. They have developed a computational model that accounts for various resistance mechanisms to weakly basic drugs. They found that a combination of mechanisms including active transport of drugs into endosomes, increased endosomal turnover, decreased endosomal pH and increased cell membrane pH gradient would yield a drug-resistant phenotype. An example of cells which show such pattern is MCF-7 cells that are chemoresistant can lower cytosolic concentrations of weak base drug such as mitoxantrone (Raghunand et al., 1999).

The regulation of cellular pH on drug resistance is further proven by another study which discovered that upregulated proton pump gene expression in cisplatin-resistant cell lines which resulted in intracellular alkalization and elevated intracellular pH in the cells. As DNA-cisplatin adducts are more easily formed under acidic conditions, the increase in the cellular pH could attenuate its cytotoxicity effect and induce drug resistance, and this was shown when proton pump inhibitor synergistically potentiated the cisplatin cytoxicity (Murakami et al., 2001). Whether or not V-ATPases are overexpressed in endosomal membrane or ILVs was not investigated by Murakami et al. (2001). Studies were conducted to investigate the effect of V-ATPase inhibitors on sensitizing chemoresistant cells (Murakami et al., 2001; Kiyoshima et al., 2013). The treatment resulted in lower acidification of vesicular organelles (detected using acridine orange stain) and increased apoptosis in three of four OSCC cell lines, indicating that vesicular acidification is an important survival mechanism of OSCC cells (Kiyoshima et al., 2013). This further points to the possibility of improving cytotoxicity of drugs such as cisplatin by combining it with proton pump inhibitors. However, it is not well understood if alkalization of vesicles will affect uptake or efflux of chemotherapy drugs.

##### Anti-Apoptotic Signaling

Apoptosis is one form of programmed cell death initiated by apoptotic proteins such as caspases, Bcl-2-associated proteins and cytochrome C. Malignant tumors are often marked with the overexpression of anti-apoptotic proteins or signals. Current molecular markers that hold prognostic and predictive values are still incapable of resolving the heterogeneity and complexity of OSCC (Chung et al., 2004). Molecular profiling by gene arrays, tissue microarrays, immunohistochemistry profiling and cluster analysis have been the standard of molecular characterization of tumors.

A study conducted a molecular profiling of 171 OSCC cases via immunohistochemical analysis and ordered them by hierarchical clustering and the expression profiles of 23 anti-apoptotic proteins were investigated. Despite being able to divide the OSCC cases into two groups—apoptotic and anti-apoptotic, there was no association between these groups with clinical and pathological characteristics such as the overall survival of patients. Thus, there is a knowledge gap in connecting apoptosis and cancer prognosis that needs to be filled. However, the analysis did showed a group of pro-apoptotic proteins that are more prominent and could be use as targeted therapies (Coutinho-Camillo et al., 2017). The complexity of apoptosis induction and the influence of individual proteins in the apoptotic network needs more elucidation and experimental verification in order to account for the heterogeneity of OSCC tumors.

Notwithstanding the current lack of understanding between apoptosis and cancer survival, apoptotic inhibition is still a chemoresistance trait that needs to be studied in depth. It has been suggested that exosomes can promote anti-apoptotic signals in both donor and recipient cells. For instance, Caspase-3, a protein known as an executioner caspase is responsible for cleaving cellular substrates that are vital to cell survival, leading to membrane blebbing and disruption of cytoskeletal functions. The release of caspase-3 containing membrane vesicles is thought to facilitate removal of apoptotic proteins in order to promote cell survival (Böing et al., 2013).

Exosomes can transduce anti-apoptotic signals to recipient tumor cells via different mechanisms. They can stimulate recipient cells via signal transduction facilitated by surface receptors to activate anti-apoptotic pathways (Xu et al., 1998; Roccaro et al., 2013). Exosomes can transfer receptors such as CD41 to target cells to trigger integrin-mediated inhibition of apoptosis by preventing cell detachment from the extracellular matrix (Boudreau et al., 1996; Wang et al., 2014). Besides that, exosomes can also transfer transcriptional factors that can induce activation of anti-apoptotic pathways (Wang et al., 2014).

As discussed above, miRNA plays an important role in mediating various signaling pathways between tumor cells as a major exosomal cargo. One of the signaling pathways include anti-apoptosis. Guo et al. showed that exosomes derived from cancer-associated fibroblasts (CAF) confers cisplatin resistance to head and neck squamous cell carcinoma (HNSCC) via exosomal miR-196a (Guo et al., 2019). HNSCC cells (CAL27 and HN4) grown on CAF conditioned media (CAF-CM) promoted cisplatin resistance and cell proliferation compared to media conditioned from chemoresistant HN4 cells (Guo et al., 2019). Physical removal of exosomes from CAF-CM by ultracentrifugation and blocking exosome release via the inhibitors GW4869 reduced the ability of CAF-CM to promote HNSCC cell survival (Guo et al., 2019). With exosomes proven as the mode of delivery for cisplatin-resistance promoting factors, miRNA array analysis of cisplatin treated CAF-derived exosomes was carried out. The test revealed a marked increase in miR-196a content (Guo et al., 2019). Subsequent MTT and colony formation assays showed that miR-196a overexpression enhanced the growth of CAL27 and HN4 HNSCC cells transfected with miR-196a (Guo et al., 2019). The target genes that interact with miR-196a were identified via miRecords algorithm to be CDKN1B and ING5. RT-PCR revealed that both genes were inhibited by miR-196a overexpression (Guo et al., 2019). ING5 is a tumor suppressor protein that inhibits cell growth and apoptosis (Gao and Han, 2018), whereas CDKN1B is a cell cycle inhibitor that slows down the progression of cell division (Cusan et al., 2018). Although the study was conducted on HNSCC cells and not specifically OSCC, it is worth noting the effects of CAF-derived exosomes on squamous cell carcinomas. Moreover, it provides further evidence of the tumor microenvironment playing an important role in chemoresistance and anti-apoptotic phenotype of tumor cells via delivery of exosomes.

Aside from exosomal enrichment of miRNAs conferring chemoresistance traits, miRNAs conferring chemosensitivity will be downregulated in resistant OSCC cells (Kulkarni et al., 2020). demonstrated that miR-30a was found to be significantly reduced in exosomes isolated from serum of OSCC patients, especially post cisplatin treatment. They also identified Beclin1 (autophagic marker) to be a binding target of miR-30a and are overexpressed in cisplatin-resistant OSCC cells. Exosomes from cisplatin-resistant cells that have been transfected with miR-30a mimics, when delivered to naïve cisplatin-resistant cells, caused a significant downregulation in Beclin1 and Bcl2 (antiapoptotic marker) expression, resulting in the sensitization of cisplatin-resistant OSCC cells. Thus, this proves that downregulation of exosomal miR-30a is a chemoresistance trait in cisplatin-resistant OSCC cells.

These experiments provide extensive evidence on the role of exosomes in propagating anti-apoptotic signals, be it from neighboring OSCC cells or from the tumor microenvironment. Based on the above studies, the main exosomal content carrying apoptotic signals are found to be miRNAs. However, the role of other exosomal contents in mediating anti-apoptotic signals should also be investigated. This is important as Coutinho-Camillo et al. (2017) had demonstrated that the pathological and prognosis characteristics of OSCC cases cannot be predicted even with the expression profiling of the complete range of apoptosis-related proteins to date. It is possible that other exosomal contents may modulate apoptosis in mechanisms unknown to us.

##### Modulation of DNA Damage Repair (DDR) mechanisms.

**FIGURE 4:**
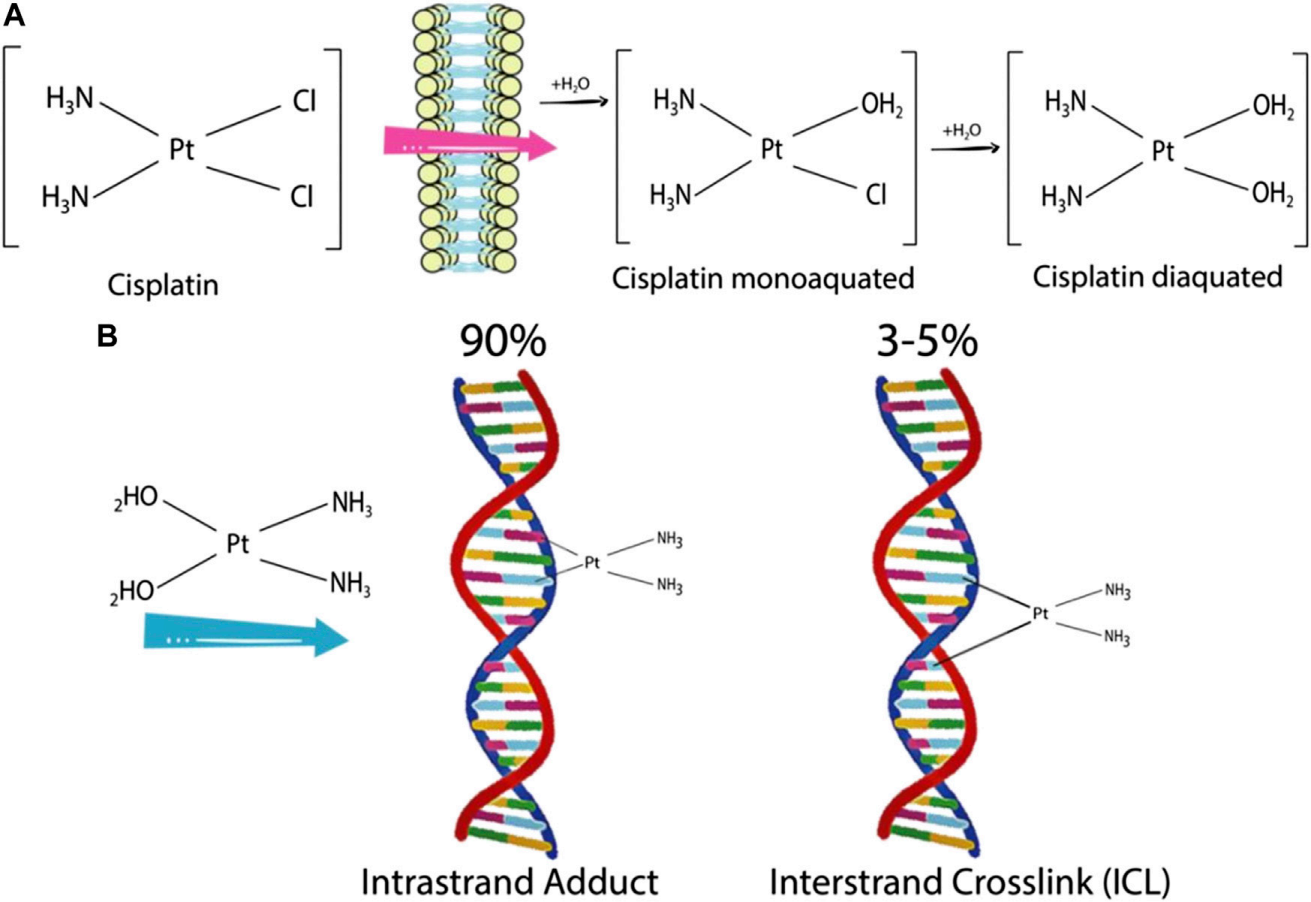
Cisplatin activation and formation of cisplatin-DNA adducts **(A)** Cisplatin is mono- and diaquated upon entering a cell. **(B)** Cisplatin can form covalent bonds with DNA bases. An intra-strand adduct is formed when cisplatin cross links two bases of the same strand, whereas an inter-strand adduct is formed when cisplatin cross links two bases from different strands. The percentages indicate the frequency of each type of DNA lesion induced by cisplatin (Rocha et al., 2018).

DNA damage repair (DDR) mechanisms play an essential role in anticancer drug resistance. Drugs such as cisplatin and carboplatin intercalate between DNA bases and introduce inter- and intra-strand linkages. These effects result in the inhibition of DNA synthesis, triggering DNA damage response and inducing apoptosis (Figure 4). The human DDR mechanism is regulated by various proteins responsible in recognition of DNA lesions, nucleotide excision and DNA ligation (Rocha et al., 2018). Exosomes play a role in modulating the DDR mechanism in cells under stress. Akiko et al. (2017) demonstrated that exosomes maintain cellular homeostasis by excreting harmful cytoplasmic DNA from cells (Akiko et al., 2017). Secretion of exosomes containing cytoplasmic DNA prevents aberrant activation of the DDR pathway, which is a precondition of apoptosis. The DDR response consists of checkpoint mechanisms that arrest cell cycle to allow the repair of damaged DNA, and if the severity is high, induce cell death via apoptosis.

Cisplatin and many chemotherapeutic drugs are known for its ability to induce apoptosis via DDR (Rocha et al., 2018). Akiko et al. (2017) observed that exosomes released from human diploid fibroblasts (HDFs) have the potential to activate the DDR pathway in recipient pre-senescent HDFs depending on the amount of exosomes present (Akiko et al., 2017). Immuno-gold labeling of double-stranded DNA (dsDNA) along with transmission electron microscopy revealed the presence of DNA in the exosomes of multivesicular bodies (MVBs). Subsequent DNA sequencing analysis points toward the origin of these dsDNA fragments being chromosomal and not mitochondrial. Thus, it was concluded that the DNA fragments released via exosomes are harmful constituents in cells. Moreover, the amount of DNA in the exosomes was enriched when pre-senescent HDFs were treated with DNA-damaging agents such as doxorubicin (Akiko et al., 2017).

Interestingly, cisplatin-induced DNA lesions that stall replication forks can result in the formation of DNA double-stranded breaks (DSBs), resulting in chromosomal DNA fragments (Rocha et al., 2018). It can thus be hypothesized that the release of chromosomal DNA-containing exosomes is a mechanism reducing DDR activation which in the context of cancer cells, promotes cancer cell survival. It is possible that a similar mechanism as stated above is utilized by OSCC cells to reduce aberrant DDR mechanisms and reducing the cell’s likelihood of undergoing apoptosis. However, similar studies on OSCC cell lines are needed to verify this. Mutschelknaus et al. (2016) discovered that tumor-derived exosomes were able to increase radioresistance in head and neck SCC (HNSCC) cells via increasing DSB repair (Mutschelknaus et al., 2016).

Although chemotherapy and radiotherapy are distinct from each other, both therapies induce DNA damage. Moreover, it has been demonstrated beforehand that exosomes from breast cancer cells can alter the phosphorylation status of DDR proteins (Dutta et al., 2014). Exosomes from irradiated and non-irradiated HNSCC BHY cells were transferred to irradiated BHY cells (Mutschelknaus et al., 2016). The number of DSB repair foci in the recipient BHY cells was analyzed and quantified after 6 h (Mutschelknaus et al., 2016). It was revealed that the number of repair foci was lower in the cells incubated with exosomes isolated from irradiated BHY cells, suggesting a quicker rate of DNA repair induced from these exosomes (Mutschelknaus et al., 2016). A similar phenomenon occurred in another HNSCC cell line FaDu (Mutschelknaus et al., 2016). Furthermore, exosomal release and uptake in HNSCC cells were shown to be higher under irradiation (Mutschelknaus et al., 2016), suggesting a putative mechanism involving exosomes which is triggered when HNSCC cells undergo DNA-damaging stress. It is important to note however, that (Mutschelknaus et al., 2016) did not investigate the exosomal content responsible in promoting the DSB repair.

DNA lesions resulting from damage induced by cisplatin and its analogues are repaired mainly via the nucleotide excision repair (NER) pathway (Rocha et al., 2018). However, a separate study conducted by Kothandapani et al. revealed that a different DDR pathway known as base excision repair (BER) is capable of mediating cisplatin cytotoxicity (Kothandapani et al., 2011). Using ovarian carcinoma cells, breast cancer cells and HeLa cells, they demonstrated that BER maintains cytotoxicity of cisplatin by competing with the productive NER pathway (Kothandapani et al., 2011). One of the proteins facilitating the BER pathway is apurinic/apyrimidinic endonuclease 1 (APE1). APE1, a protein shown to be elevated in blood serum of OSCC patients (Yousafzai et al., 2018). Whether or not the protein in blood serum originates from exosomes remains unknown. However, Nath et al. (2017) demonstrated that APE1 is released from monocytic cells via exosomes (Nath et al., 2017). The presence of APE1 in isolated exosomes was examined by western blot analysis which showed that APE1 levels increased significantly in secreted exosomes compared to whole cell extract of the monocytic cells (Nath et al., 2017). Thus, it is possible that cell release of APE1 via exosomes is a chemoresistance mechanism inhibiting the BER pathway, allowing OSCC cells to respond to cisplatin-induced DNA damage via NER. However, this line of thought would need more experimental investigation based on OSCC models.

In order to prove the role of exosomes in modulating DDR mechanisms in chemoresistant OSCC, more studies establishing a direct link are needed. To date, there is only evidence showing the involvement of OSCC exosomes in mediating DNA repair, but none in a chemoresistance context.

##### Immunomodulation by Exosomes

Aside from modulating DDR mechanisms, exosomes have been shown to possess immunomodulatory effects (Momen-Heravi and Bala, 2018; Cai et al., 2019). Cellular crosstalk between tumor cells and their microenvironment which consists of immune cells such as monocytes and macrophages is important in tumor progression. Chemotherapeutic drugs such as cisplatin and doxorubicin inhibit DNA synthesis, which happens frequently in dividing cells. Thus, dividing immune cells often become targets of these drugs as well, weakening a patient’s immune response against tumors. Moreover, tumor cells under chemotherapy-induced stress are capable of enhancing their immunosuppression abilities toward immune cells in the surrounding microenvironment. Baghdadi et al. demonstrated that Interleukin 34 (IL34) was produced by cancer cells under chemotherapy-induced stress to enhance local immunosuppression of tumor-associated macrophages (TAMs), increasing the ability of cancer cells to evade an immune response (Baghdadi et al., 2016).

A well investigated pathway which mediates the critical changes characteristic of innate and adaptive immune responses is the nuclear factor kappa-light-chain-enhancer of activated B cells (NF-κB) pathway (Hayden et al., 2006). NF-κB is a family composed of five related transcription factors: p50, p52, RelA, c-Rel and RelB (Gilmore, 2006). These transcription factors possess a N-terminal DNA-binding domain that binds to target DNA sequences called κB sites to regulate gene expression (Jiang et al., 2009). In a study conducted by Momen-Heravi and Bala (2018), exosomes from OSCC cells can reprogram monocytes via the NF-κB pathway (Momen-Heravi and Bala, 2018). They discovered that exosomes released from CAL27 cells both in normal and under ethanol conditions contained a high signal of miR-21 (Momen-Heravi and Bala, 2018). Exosomes released from THP1 monocytes however, does not contain a similarly high level of miR-21, implying a specific sorting mechanism involved in packaging of miR-21 into exosomes in OSCC cells (Momen-Heravi and Bala, 2018). Fluorescence-labelled exosomes from CAL27 cells were observed to be taken up by THP1 monocytes (Momen-Heravi and Bala, 2018). This was further proven by the observation that Cytochalasin D (an inhibitor of endocytic pathways and exosome uptake) treatment resulted in decrease of miR-21 level in THP1 monocytes (Momen-Heravi and Bala, 2018). Electrophoretic mobility shift assay (EMSA) of specific NF-κB consensus sequences showed activation of NF-κB p50 heterodimers in THP1 monocytes (Momen-Heravi and Bala, 2018). It was postulated that exosomes released from CAL27 were capable of reprogramming the monocytes. Monocyte chemoattractant protein-1 (MCP1) which molds the tumor microenvironment and promotes tumorigenesis (Yoshimura, 2017) was shown to increase significantly in recipient THP1 monocytes after co-culture of exosomes derived from CAL27 (Momen-Heravi and Bala, 2018). The level of other pro-oncogenic factors such as matrix metallopeptidase 9 (MMP9), cyclooxygenase-2 (COX2) mRNA levels, vascular endothelial growth factor (VEGF) and interleukin-6 (IL6) were increased after co-culture with exosomes (Momen-Heravi and Bala, 2018). However, preliminary size characterization of the EVs released from CAL27 cells via nanoparticle tracking analysis revealed that there is a mix of both exosomes and microvesicles. The inference that THP1 monocytes are reprogrammed by the microvesicles carrying miR-21 is also viable. Notwithstanding\, these observations still support the conclusion that exosomes from OSCC cells are capable of modulating the immune function of surrounding monocytes and establishing a pro-inflammatory milieu via miR-21 (Momen-Heravi and Bala, 2018). Chronic inflammation promotes tumorigenesis by disabling the immune system from attacking tumor cells, inducing cell proliferation and genetic instability leading to oncogenic mutations (Xia et al., 2014).

Aside from monocytes, exosomes from OSCC can also reprogram macrophages. Cai et al. (2019) demonstrated that OSCC-derived exosomes containing miR-29a-3p promote M2 subtype macrophage polarization (Cai et al., 2019). Exosomes isolated from SCC9 and CAL27 OSCC cells were co-cultured with macrophages (Cai et al., 2019). The expression level of M2 subtype macrophage marker proteins such as CD163, CD206, Arg-1 and IL-10 increased after co-culturing (Cai et al., 2019). After that, the conditioned-medium of co-culturing exosomes and macrophages was used to culture SCC9 and CAL27 cells (Cai et al., 2019). Transwell assay conducted on SCC9 and CAL27 showed an increase in cell invasion and proliferation, proving that the M2 subtype macrophage polarization promotes tumorigenesis and pro-metastatic environment for OSCC cells (Cai et al., 2019). These results were also supported by Kazumasa et al. (Mori et al., 2011). They performed immunohistochemical analysis to identify M2 TAMs in surgically resected OSCC specimens from 50 patients and discovered a positive correlation between the proportion of M2 TAMs and the pathological grade of the OSCC specimen (Mori et al., 2011). Thus, it can be concluded that immunomodulation mediated by exosomes is an important topic of investigation and should be included in the search for therapeutic strategies against OSCC chemoresistance.

##### Epithelial-Mesenchymal Transition (EMT)

**FIGURE 5:**
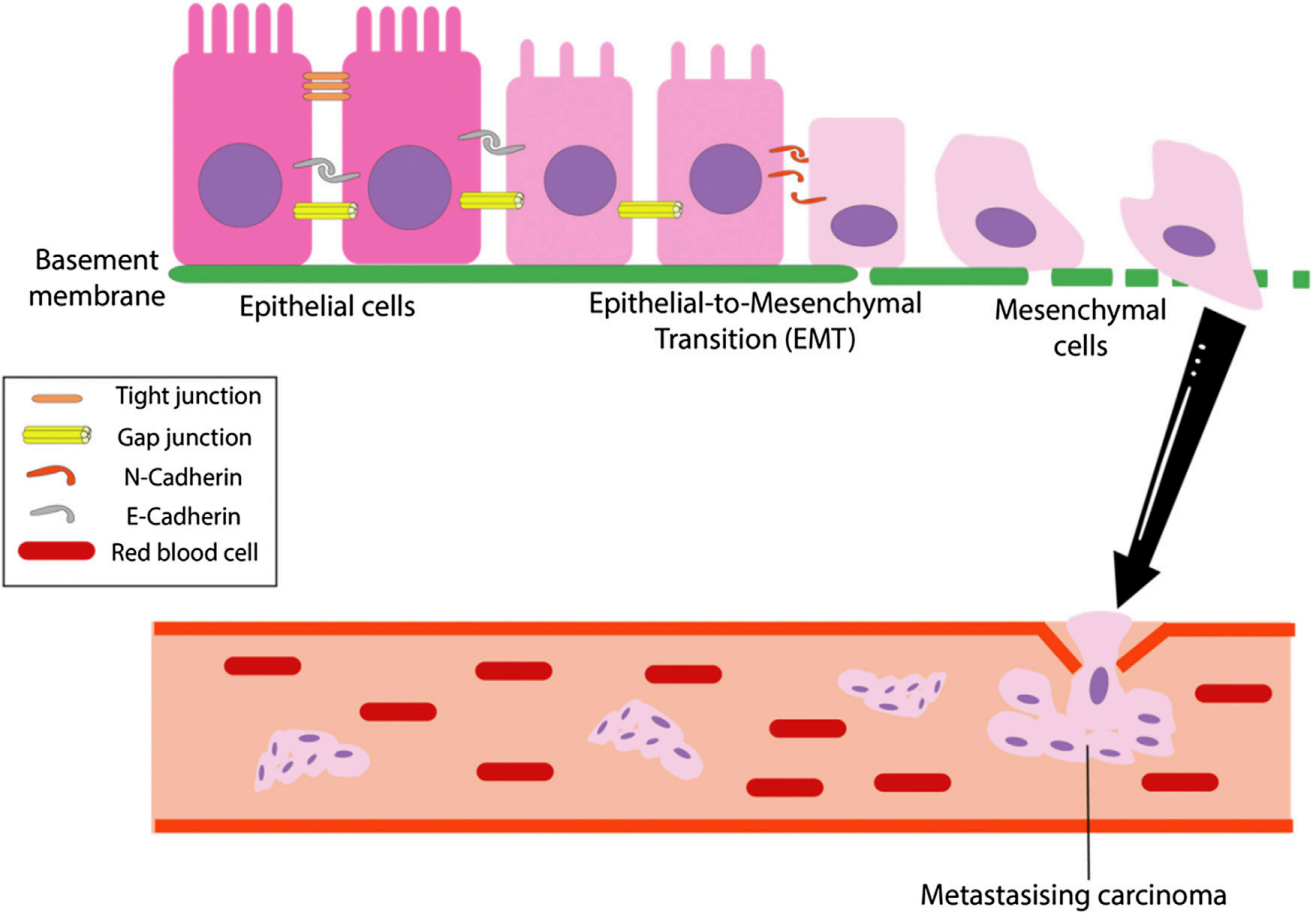
The overall EMT process. The epithelial-mesenchymal transition (EMT) is the process which tumor cells change from epithelioid to mesenchymal cell morphology and is marked by a loss of cell polarity, tight, gap and adherent junctions in epithelial cells, resulting in mesenchymal cells that has increased migratory and invasive capabilities (Li et al., 2019).

The epithelial-mesenchymal transition (EMT) is a conserved process marked by the loss of epithelial characteristics and gain of mesenchymal phenotype of a cell at a genetic, epigenetic and morphological level (Greening et al., 2015). It is characterized by the downregulation of cell adhesion molecules E-cadherin and β-catenin with the concomitant upregulation of mesenchymal markers N-cadherin and vimentin (Thomas et al., 2018). EMT was initially thought to be confined within embryological development before its conception as a mechanism endowing metastatic and invasive properties to a tumor (Greening et al., 2015). During EMT, the epithelial cells lose apical-basal polarity and epithelial cell-cell contacts such as tight junctions, adherens junctions and the actin cytoskeletal architecture are disassembled and reorganized (Lu and Kang, 2019). The resulting mesenchymal cells attain a spindle-shape morphology and gain motility. The cellular changes are accompanied by a change in the expression of epithelial genes. For instance, cells undergoing EMT will express matrix metalloproteinases (MMPs) to degrade and invade the basal extracellular matrix (Lu and Kang, 2019). Figure 5 below briefly outlines the main steps of the EMT process.

There is increasing evidence that suggests EMT contributes to chemoresistance. Earlier studies have established the connection between transcription factors regulating expression of EMT genes and drug sensitivity (Vega et al., 2004; Arumugam et al., 2009; Kurrey et al., 2009). The studies demonstrated an increase in drug sensitivity when the transcription factors are repressed. Recent studies utilizing murine models of pancreatic and breast cancers demonstrate that EMT is dispensable for metastasis to occur, but contributes strongly to chemoresistance (Xiaofeng et al., 2015; Shields, 2018). Clinicopathological studies and immunohistochemical analysis of OSCC EMT markers have also supported the idea that EMT is not a necessary step for metastasis, but may be responsible for other properties such as chemoresistance and the formation of stem cell-like properties (Tarin et al., 2005; Chui, 2013; Zidar et al., 2018).

EMT can be mediated by exosomes in OSCC cells undergoing hypoxic stress. Hypoxia has often been postulated to act as an environmental pressure resulting in malignant evolution of tumor cells (Lu and Kang, 2010). The metastatic process of tumors under hypoxia was accelerated via EMT (Lu and Kang, 2010; Li et al., 2016). Li et al. demonstrated that hypoxic OSCC cells deliver miR-21 via exosomes to induce EMT in normoxic cells (Li et al., 2016). Immunoblotting for CD63 revealed that hypoxia induced exosome release from CAL27 and SCC9 OSCC cell lines (Li et al., 2016). These exosomes were isolated and incubated along with normoxic cells. The migration and invasion of the cells measured using wound healing assays increased (Li et al., 2016). This along with the increased expression of EMT marker vimentin proves that exosomes from hypoxic OSCC cells can induce EMT in recipient normoxic cells (Li et al., 2016). MiRNA expression profile and RT-PCR revealed that miR-21 was expressed higher in hypoxic exosomes compared to normoxic exosomes (Li et al., 2016). Hence, Li et al. (2016) concluded that miR-21 was the main mediator inducing EMT changes in normoxic cells from hypoxic cells (Li et al., 2016). This theory was further validated by murine xenograft models, where injection of hypoxic exosomes into the xenograft tumors increased the growth, weight of tumor and caused an overexpression of miR-21 compared to normoxic exosomes (Li et al., 2016).

A recent study conducted by (Kirave et al., 2020) further elucidated the EMT modulatory properties of OSCC exosomes. In this study, it was shown that exosomes from cisplatin-resistant OSCC cells carrying miR-155 are capable of inducing miR-155 overexpression in sensitive OSCC cells. The binding target of miR-155 was verified via bioinformatics and transfection experiments to be FOXO3, thereby inhibition of this protein leads to upregulation of EMT markers. Furthermore, exosomes derived from cisplatin-resistant cells transfected with miR-155 mimics are shown to be enriched in miR-155. When sensitive OSCC cells are treated with these exosomes, protein and mRNA levels of FOXO3 decreased and cisplatin resistance of the cells were enhanced. These results proved a close link between EMT and chemoresistance, and the important role played by exosomes in facilitating EMT in OSCC.

Besides exosomal miR-21 and miR-155, other EMT-modulating factors which may be potential cargoes of OSCC exosomes are epidermal growth factor (EGF), CD47, miR-222 and other EMT transcription factors (EMT-TFs) (Nieto et al., 2016; Ouyang et al., 2017; Fujiwara et al., 2018; Pai et al., 2019). A separate study done by Chang et al. showed that the miRNA let-7d, a known tumor suppressor was repressed as EMT-promoting transcription factors Twist and Snail are overexpressed in OSCC (Chang et al., 2011). Cell viability experiments showed that ectopic overexpression of let-7d suppressed the chemoresistance of OSCC cells to cisplatin and paclitaxel (Chang et al., 2011). EMT-TFs are known to be exosomal cargoes (Nieto et al., 2016). Thus, it is possible that EMT-TFs can be transported as exosomal cargoes to distant cells and promote EMT and chemoresistance phenotypes. However, future experiments are needed to establish the presence of these EMT-TFs in OSCC exosomes and investigate their effects on recipient cells.

##### Exosomes From *Cancer* Stem Cells

The cancer stem cell (CSC) hypothesis postulates that CSCs are responsible for the maintenance and recurrence of tumors (Yoo and Kwon, 2015). This small subset of cancer cells possess properties such as self-renewal, slow replication, intrinsic resistance to chemotherapy and an ability to give rise to differentiated progeny (O’flaherty et al., 2012). Cellular dormancy allows CSCs to escape antineoplastic treatment as they are recruited to the anti-mitotic and quiescent G0-phase (Kleffel and Schatton, 2013). A lower proliferation rate would reduce the efficacy of DNA-damaging chemotherapeutic agents. Several studies of other cancer models have shown that exosomes are capable of conferring stemness to cancer cells (Koch et al., 2014; Bliss et al., 2016). Mesenchymal stem cells (MSCs)-derived exosomes containing miR-222 induced quiescence in breast cancer cells *in vitro* and *in vivo*, thus conferring drug resistance (Bliss et al., 2016). In an *in vitro* model of diffuse large B-cell lymphoma (DLBCL), exosomes containing Wingless-related integration site (Wnt) signaling factor from DLBCL can induce a CSC phenotype in recipient cells (Koch et al., 2014). CSCs are able to endure environmental stress such as radiation and hypoxia by maintaining their stemness via exosomes containing Hedgehog, Wnt, β-catenin and other mRNAs and proteins (Fatima and Nawaz, 2015). These exosomal cargoes can sustain the self-renewal and clonogenic properties of CSCs.

CSC-like phenotype can be induced by microRNA such as miR-146a (Roychoudhury et al., 2018). Roychoudhury et al. demonstrated that miR-146a overexpression enhances the stemness of OSCC cells by augmenting CD44 and CD24 levels (Roychoudhury et al., 2018). CD24 was identified as a functional target of miR-146a (Roychoudhury et al., 2018). Roychoudhury et al. (2018) demonstrated that miR-146a confers stemness via suppression of CD24. Mechanistic analysis revealed that higher CD24 levels inhibit AKT phosphorylation leading to degradation of β-catenin, an important factor for cancer stemness (Roychoudhury et al., 2018). However, studies to verify whether exosomes isolated from OSCC or the surrounding population of CSCs contain miR-146a are needed. Whether or not exosomes play a role in conferring cancer stemness in OSCC remains an unsolved mystery.

### Microvesicles

Microvesicles are heterogenous, membranous sacs that are shed directly from the plasma membrane of a cell (Raposo and Stoorvogel, 2013). They are a distinct group of extracellular vesicles that are much larger in size (100–1,000 nm) compared to exosomes. Like exosomes, they also carry various forms of cargo including proteins, miRNA and RNA transcripts.

The biogenesis of microvesicles involves the outward blebbing and pinching of the plasma membrane. The process is accompanied by localized changes in the plasma membrane protein and lipid components which are involved in modulating membrane curvature (McMahon and Boucrot, 2015). Synergistic action of multiple mechanisms such as asymmetric partitioning of transmembrane domains, protein scaffolding and cytoskeletal reorganization both at the nanoscopic and macroscopic level mediate microvesicle budding (Harvey and Jennifer, 2005). Due to the heterogeneity of their size, microvesicles can be generated by various distinct mechanisms that may partially overlap with those of exosome biogenesis. For example, sphingomyelinases that convert sphingomyelin to ceramide are shown to be both responsible in MVB formation and the budding of microvesicles (Trajkovic et al., 2008; Bianco et al., 2009).

While it has not been extensively studied, microvesicles have been shown to modulate drug resistance in cancer cells, making them another factor to consider in combating drug resistance. Microvesicles isolated from OSCC have been shown to be larger and enriched than normal control cells. Their cargo contains oncogenic miRNA such as miR-21, miR-27a, miR-27b, and miR-155 (Momen-Heravi and Bala, 2018). One of them, miR-21, has been strongly evidenced to contribute toward OSCC chemoresistance via the STAT3/miR-21 axis (Xuan et al., 2014). Besides that, the miRNA signatures have been evidenced to induce pro-inflammatory phenotype in the tumor microenvironment by reprogramming monocytes via the NF-κB pathway (Momen-Heravi and Bala, 2018).

### Microvesicle-Mediated Chemoresistance

The involvement of microvesicles (MVs) in chemoresistance properties are not as extensive as exosomes. However, they have been implicated in intercellular communication within the tumor microenvironment such as stromal and immune cells, transport of drug efflux proteins, drug sequestration and anti-apoptotic signaling (Kim et al., 2005; Abid Hussein et al., 2007; Gong et al., 2013; Zhang et al., 2014a; Zhang et al., 2014b). The pathway involved for the microvesicle-mediated chemoresistance is through the cargoes that is packed into MVs. Transmembrane, soluble proteins, lipids or nucleic acids that are packed into MVs shedding from plasma membrane could be endocytosed by recipient cells and results in the delivery of its cargoes into the recipient cells, thus transferring the information and influence the cancer development (Bian et al., 2019).

MVs isolated from A2780 ovarian cancer cells were shown to carry P-glycoprotein (P-gp), a protein overexpressed in multidrug resistance (MDR) phenotype (Zhang et al., 2014a). Intercellular transfer of MVs mediate the ‘sharing’ of P-gp which confers paclitaxel-resistance to recipient A2780 cells. Aside from that, MVs have also been shown to sequester drugs and reduce the cytosolic free drug concentration in breast adenocarcinoma and acute lymphoblastic leukemia cells (Gong et al., 2013). MVs have also shown to carry anti-apoptotic potential. A study conducted by Abid Hussein et al. demonstrated that inhibiting the release of caspase-3-containing MVs triggers endothelial cell detachment and subsequent apoptosis, thus showing that release of caspase-3-containing MVs is an important anti-apoptotic mechanism (Abid Hussein et al., 2007). In addition, MV-carrying EGFR can be taken up by endothelial cells which in turn activates MAPK and Akt pathways, this will lead to the endogenous expression of VEGF, followed by the activation of VEGF receptor-2 and tumor angiogenesis (Al-Nedawi et al., 2009).

Whether or not MVs of OSCC carry out similar functions is yet to be investigated. On the other hand, MVs from OSCC have already been proven to induce apoptosis of T lymphocytes (Kim et al., 2005). OSCC patients often have a high proportion of T lymphocytes undergoing apoptosis (Kim et al., 2005). T cells found at tumor sites often experience dysregulation of signaling factors such as TCR-ζ downregulation (Reichert et al., 2002). Kim et al. (2005) demonstrated that Fas Ligand (FasL)-Positive MVs isolated from the sera of OSCC patients can induce apoptosis of T lymphocytes, enabling the OSCC cells with immunosuppression capabilities (Kim et al., 2005). Aside from that, Momen-Heravi and Bala (2018) demonstrated that MVs from CAL27 cells carrying miR-21 are capable of reprogramming monocytes via the NF-κB pathway (Momen-Heravi and Bala, 2018). However as stated above, the EVs isolated from Momen-Heravi et al. contain a mixture of exosomes and MVs (Momen-Heravi and Bala, 2018).

The involvement of MVs in facilitating intercellular communication, changes in tumor microenvironment and drug resistance in OSCC needs more investigation. Besides that, improvements and optimisation of isolation protocols and methodologies are of paramount importance. There is a need to achieve an EV subtype yield with the highest purity and integrity in order to avoid studying MVs and exosomes in the same context. Characterization methods of EVs need continual improvement in order to distinguish between MVs and exosomes. The distinguishing of MVs and exosomes from each other will avoid wrongly attributing EV cargoes and signaling pathways to an EV subtype.

## Conclusion

The involvement of EVs in mediating chemoresistance in OSCC still requires ample investigation. To date, only a handful of direct links implicating EV involvement in OSCC chemoresistance have been established. Nevertheless, these findings provide sufficient basis that both exosomes and microvesicles play an important role in several chemoresistance mechanisms. In this review, we have discussed several roles of EVs in the regulation of chemoresistance in OSCC including its exosomal content, drug efflux by EVs, alteration of vesicular pH, anti-apoptotic signaling transmitted by EVs, modulation of DDR mechanisms, immunomodulation by exosomes, transport of EMT promoting genes and lastly, microvesicle-mediated chemoresistance.

In summation of the above findings related to exosomes, exosomal content such as miR-21 and miR-24 are recognized as key oncogenic factors mediating OSCC cell survival. Aside from that, drug efflux by EVs for instance cisplatin efflux mediated by copper transporters ATP7A and ATP7B remains an active field of investigation. Novel studies suggest that cisplatin may be transported out by ATP7B via lysosomal exocytosis, albeit similar studies done on OSCC are yet to be made. Alteration of vesicular pH by vacuolar ATPases residing on the endomembrane presents a viable mechanism of OSCC chemoresistance as cisplatin sensitivity depends on the acidity of intracellular environment.

Besides that, exosomes from cancer-associated fibroblasts can deliver anti-apoptotic signaling miRNAs that alter expression of genes regulating apoptosis. In addition, exosomal release of chromosomal DNA may be a potential mechanism utilized by OSCC to reduce aberrant DDR activation and apoptosis. Furthermore, exosomes from OSCC have been shown to be capable in reprogramming monocytes via the NF-κB pathway and macrophages via miR-29a-3p, thus mediating immunosuppression of the tumor microenvironment. Aside from that, an emerging perspective suggests that epithelial-to-mesenchymal transition is a chemoresistant mechanism in addition to being a metastatic process. Exosomes from OSCC cells carrying EMT-promoting cargoes such as miR-21, miR-155 (experimentally proven to confer chemoresistance) and EMT-transcription factors may confer chemoresistance phenotypes to recipient cells. Last but not least, exosomal miR-146a are implicated in enhancing stemness in OSCC cells. *Cancer* stem cells are known for self-renewal and low proliferation properties, thus making them harder targets for DNA-damaging drugs. Findings in relation to microvesicle-mediated chemoresistance in OSCC however, are limited. Nevertheless, notable findings of MV-mediated chemoresistance include apoptosis induction of T lymphocytes and reprogramming of monocytes by OSCC MVs. However, it is important that EV characterization methods be standardized across laboratories to distinguish MVs from exosomes.

In addition to that, the various pathways of biogenesis and endocytic mechanisms of EVs need to be mapped in order for EV inhibition to be considered as a synergistic complement to chemotherapy. Current research has no guarantee that EV inhibition will fully cripple tumor progression. In addition, due to the heterogeneity of OSCC cases, there is no guarantee that a study done on a few OSCC cell lines are fully applicable to all OSCC cases. Current molecular diagnostics utilizing tumor biomarkers are unable to accurately predict tumor evolution and behavior due to intra- and inter-tumor heterogeneity (Ogino et al., 2012). The complex interactions between tumor cells and the host cells, which is influenced by genomic variation and environmental factors such as diet and lifestyle presents a longstanding impasse in clinical diagnostics and treatment strategies. With that in mind, it is of paramount importance that deeper studies probing into the mechanisms of EV-mediated chemoresistance are carried out to improve the resolution of molecular characterizations on OSCC cases.
